# Activated and inactivated immune responses in *Caenorhabditis elegans* against *Photorhabdus luminescens* TT01

**DOI:** 10.1186/2193-1801-3-274

**Published:** 2014-06-01

**Authors:** Kazuki Sato, Toyoshi Yoshiga, Koichi Hasegawa

**Affiliations:** Laboratory of Nematology, Department of Applied Biological Sciences, Saga University, Saga, 840-8502 Japan; Laboratory of Terrestrial Microbial Ecology, Graduate School of Agriculture, Kyoto University, Sakyo, Kyoto, 606-8502 Japan; The United Graduate School of Agricultural Sciences, Kagoshima University, Kagoshima, 890-8580 Japan; Department of Environmental Biology, College of Bioscience & Biotechnology, Chubu University, 1200 Matsumoto, Kasugai, 487-8501 Japan

**Keywords:** Entomopathogenic nematode, *Photorhabdus luminescens*, *Caenorhabditis elegans*, Insulin/IGF-1, *Heterorhabditis bacteriophora*, Pathogenicity

## Abstract

The Gram-negative bacterium *Photorhabdus luminescens* which symbiotically associates with the entomopathogenic nematode *Heterorhabditis bacteriophora*, has a broad insecticidal and nematicidal activity. The virulence of *P. luminescens* toward the non-mutualistic nematode *Caenorhabditis elegans* has not been described. We showed that when fed on *P. luminescens*, the intestinal cells of *C. elegans* worms become delicate and some crystal-like structure was developed within the intestinal lumen. Next, we examined the requirement of the p38 mitogen-activated protein kinase (MAPK) and insulin/IGF-1 signaling pathway against *P. luminescens*. Depletion of *pmk-1* by RNAi enhances susceptibility to *P. luminescens*, and numerous downstream targets regulated by the p38 MAPK pathway were induced when fed on *P. luminescens*. On the other hand, knockdown of *daf-16* has no effects on *C. elegans* lifespan, but knockdown of *daf-2* dramatically increased resistance to *P. luminescens* in a *daf-16*-dependent manner. We also revealed one of the *daf-2* ligands *ins-7* was induced and *ins-7* deletion mutant survived longer when fed on *P. luminescens*. These results suggest the p38 MAPK pathway is activated and required for the host defense against *P. luminescens*. Insulin/IGF-1 signaling pathway is inactivated by *P. luminescens* through the overexpression of insulin-like gene.

**Electronic supplementary material:**

The online version of this article (doi:10.1186/2193-1801-3-274) contains supplementary material, which is available to authorized users.

## Background

*Photorhabdus luminescens* is an enteric Gram-negative bacterium which can be a pathogen producing a broad-spectrum toxins with antibacterial, antifungal insecticidal, and nematicidal activities, or a symbiont of the entomopathogenic nematode (EPN) *Heterorhabditis bacteriophora* orchestrating insect pathogenicity (Waterfield et al. 2009). A highly specialized mechanism of the bacterium-EPN association and adaptation has evolved and well established. Infective juveniles (IJs) of the EPNs invade the insect hosts and release their symbiotic bacteria into the hemocoel (Ciche and Ensign 2003). The bacteria suppress insect host defenses via production of phenoloxidase inhibitor (Eleftherianos et al. 2007), and produce insecticidal toxins to kill insect hosts within 48 hours after infection. EPNs consume bacteria and insect tissues to support their growth and reproduction. Meanwhile, the bacteria produce antibiotics and deterrent chemicals to protect the insect cadaver from microorganisms and other scavenger animals (Richardson et al. 1988; Williams et al. 2005; Gulcu et al. 2012). IJs emerge under the control of *H. bacteriophora*-producing pheromone (Noguez et al. 2012), are released from the insect cadaver carrying their aggressive symbiotic bacteria in the intestine and seek for new hosts (Ciche 2007).

Several toxin proteins produced by *P. luminescens* and other pathogenic bacteria have been identified, e.g. the toxin complex (Tc), the “Makes caterpillars floppy” (Mcf), Photox, and *Photorhabdus* insect related (Pir) toxins (Bowen et al. 1998; Daborn et al. 2002; Duchaud et al. 2003; Visschedyk et al. 2010). Even though the pathogenicity of several *P. luminescens* toxins is under active investigation (Dowling et al. 2004, 2007; ffrench-Constant et al. 2007; Vlisidou et al. 2009; Lang et al. 2010, 2011, 2013; Gatsogiannis et al. 2013; Yang and Waterfield 2013) and the genome data of *P. luminescens* are available (Duchaud et al. 2003), the underlying mechanism of their virulence remains unclear.

The mechanisms of host defense response against bacterial pathogens have been recently studied using the genetic model organism *Caenorhabditis elegans*. This approach successfully identifies the broadly conserved pathways involved in the host responses, e.g. Toll-like receptor pathway (Pujol et al. 2001; Tenor and Aballay 2008), p38 MAPK pathway (Kim et al. 2002; Troemel et al. 2006; Shivers et al. 2009, 2010), insulin/IGF-1 signaling pathway (Garsin et al. 2003; Murphy et al. 2003; Kawli and Tan 2008), and SKN-1/NRF oxidative stress pathway (van der Hoeven et al. 2011; Papp et al. 2012).

Although several studies have indicated that some *P. luminescens* strains displayed pathogenicity toward *C. elegans* (Couillault and Ewbank 2002; Sicard et al. 2007; Engelmann et al. 2011; Fischer et al. 2012, 2013; Julien-Gau et al. 2014), detailed phenotypes and the roles of host defenses have not been characterized. In this study we tested the pathogenicity of *P. luminescens* TT01, a mutualistic bacterial strain of the *H. bacteriophora* TT01 to *C. elegans*. Here we reported the phenotypes observed in *C. elegans* when infected by *P. luminescens* and revealed a similar molecular strategy with other pathogens to exert its pathogenicity.

## Results

### *P. luminescens*TT01 causes drastic damage to *C. elegans*intestine

**Figure 1:**
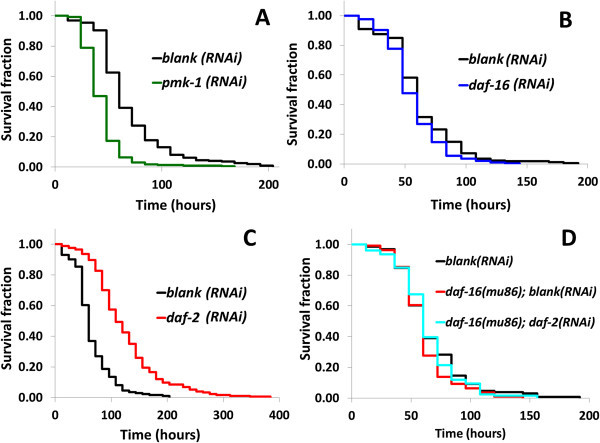
**Two pathways contribute to** ***C. elegans*** **resistance against** ***P. luminescens*** **. (A)** *pmk-1-*knocked down *C. elegans* decreased resistance against *P. luminescens* (*P* < 0.005). **(B)** *daf-16*-knocked down did not affect *C. elegans* resistance against *P. luminescens* (*P* > 0.05). **(C)** *daf-2*-knocked down *C. elegans* increased resistance against *P. luminescens* (*P* < 0.005). **(D)** Null mutation of *daf-16* did not affect *C. elegans* lifespan (*P* > 0.05), and knockdown of *daf-2* didn’t affect *daf-16* null mutant (*P* > 0.05). Survival curves are presented based on three or two trials (see Table 1). *P* values were calculated by log-rank test compared with the *blank(RNAi)*.

**Figure 2:**
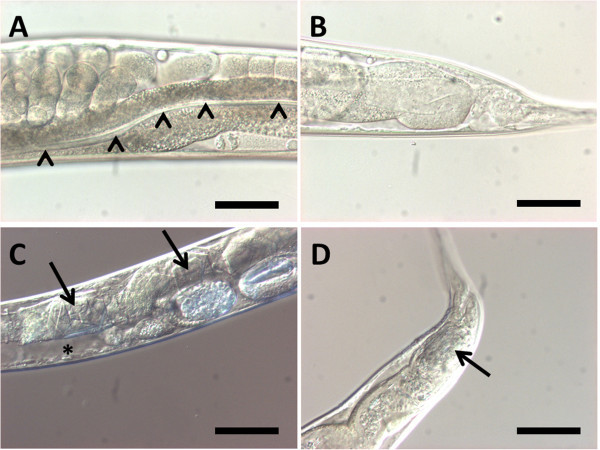
***P. luminescens*** **affects** ***C. elegans*** **intestinal cells** ***.*** **(A)** Nomarski DIC images of *C. elegans* intestine fed on *E. coli* **(A, B)** or *P. luminescens* **(C, D)** for 44 hours. **(A)** Uterus region. Arrowheads indicate healthy intestinal lumen. **(B)** Tail region. **(C)** Uterus region. Intestinal cells are delicate and pseudocoelomic space becomes larger (asterisk). Arrows indicate crystal-like structures constructed in the intestinal lumen. **(D)** Tail region. Arrow indicates crystal-like structures constructed in the intestinal lumen. Anterior is left. Scale bars, 50 μm.

*C. elegans* can be infected by many other bacterial strains (Couillault and Ewbank 2002; Sicard et al. 2007; Engelmann et al. 2011; Fischer et al. 2012, 2013; Julien-Gau et al. 2014). Here we showed that *P. luminescens* TT01 was also highly pathogenic to *C. elegans*. More than 90% of L4- staged *C. elegans* feeding on *P. luminescens* died within 5 days (Figure 1, Table 1, Blank RNAi). Body and brood size were largely reduced and the hatched larvae are developmentally retarded; most of the F1 did not develop to adulthood (data not shown). *C. elegans* intestinal cells were delicate. After 2 hours of feeding, the intestinal lumen started to swell and some crystal-like structures began to form (Figure 2A-D). This layered crystal-like structure was also observed in another bacteriovorous nematode Rhabditidae sp. (Additional file 1). Next we cultured L4 stage *C. elegans* on *P. luminescens*-seeded plates for 12 hours followed by recovery on *Escherichia coli* OP50. After 24 and 48 hour feeding on *E. coli* OP50, the crystal-like structure remained in the intestinal lumen (Table 2) and didn’t change the crystal size (Additional file 2), but the body morphology seemed healthy (Additional file 2) and reproduced normally (data not shown).

**Table 1:** **Effect of** ***P. luminescens*** **on** ***C. elegans*** **lifespan**

Trial	Genotype	Lifespan*	% increase	n	***P***value**
1	N2, *Blank(RNAi)*	63.7		39	
	N2, *pmk-1(RNAi)*	42.4	−33.4	73	< 0.005
	N2, *daf-16(RNAi)*	55.9	−12.2	47	> 0.05
	N2, *daf-2(RNAi)*	140.0	+119.8	42	< 0.005
2	N2, *Blank(RNAi)*	65.3		77	
	N2, *pmk-1(RNAi)*	48.5	−25.8	79	< 0.005
	N2, *daf-16(RNAi)*	55.6	−14.8	71	> 0.05
3	N2, *Blank(RNAi)*	75.0		81	
	N2, *pmk-1(RNAi)*	38.6	−48.5	84	< 0.005
	N2, *daf-2(RNAi)*	125.3	+67.1	51	< 0.005
4	N2, *Blank(RNAi)*	53.9		51	
	N2, *daf-16(RNAi)*	56.7	+5.2	79	> 0.05
	N2, *daf-2(RNAi)*	117.4	+118.0	75	< 0.005
5	N2, *Blank(RNAi)*	62.1		63	
	*daf-16(mu86), Blank(RNAi)*	57.5	−7.3	39	> 0.05
	*daf-16(mu86), daf-2(RNAi)*	65.4	+5.3	58	> 0.05
6	N2, *Blank(RNAi)*	72.7		68	
	*daf-16(mu86), Blank(RNAi)*	62.1	−14.6	70	> 0.05
	*daf-16(mu86), daf-2(RNAi)*	62.5	−14.1	68	> 0.05
7	N2	64.6		87	
	*ins-7(tm1907)*	76.3	+18.3	83	< 0.01
8	N2	64.0		88	
	*ins-7(tm1907)*	77.1	+20.6	75	< 0.05

*Mean lifespan (hour).

**P values of each trial were calculated by log rank test compared with controls.

**Table 2:** **Crystal-like structures do not disappear once constructed**

	Pl 12 h + Ec 0 h		Pl 12 h + Ec 48 h	
	Crystal	No crystal	Crystal	No crystal
1st Trial	10	0	20	0
2nd Trial	18	3	18	2
3rd Trial	20	0	18	2

Values indicate the number of nematodes in which the crystal-like structure was formed. Pl 12 h + Ec 0 h; *C. elegans* grown on *P. luminescens* for 12 hours from the L4 stage. Pl 12 h + Ec 48 h; *C. elegans* grown on *E. coli* for 48 hours after 12-hour incubation on *P. luminescens* from the L4 stage. The significant differences of present/absent of the crystal in each trial were calculated by Fischer’s exact test.

**Figure 3:**
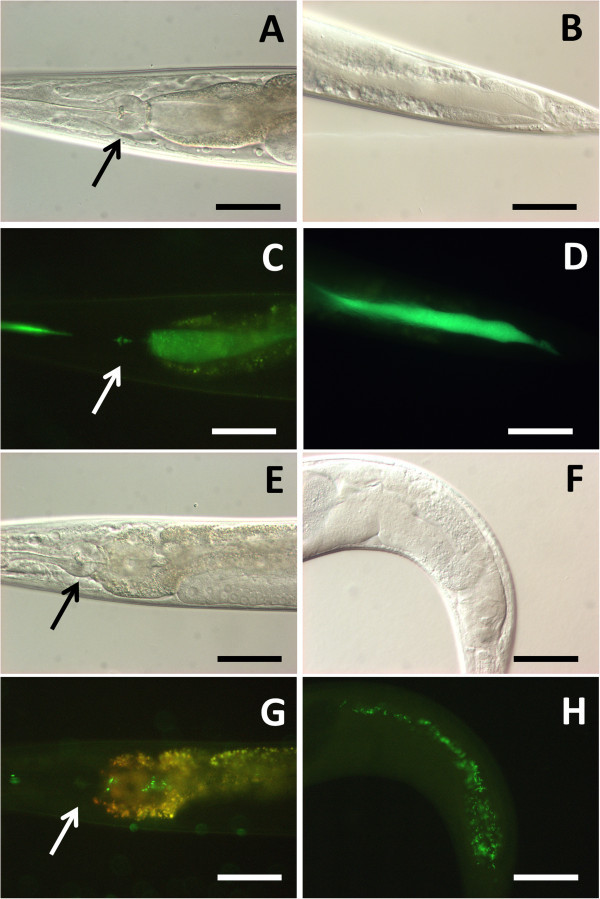
**Intact** ***P. luminescens*** **gets to the** ***C. elegans*** **intestinal lumen.** Nomarski DIC **(A, B, E, F)** or fluorescence **(C, D, G, H)** images of *C. elegans*, fed on GFP-labeled *E. coli* **(A-D)** or GFP-labeled *P. luminescens* **(E-H)** for 24 hours. Intestinal lumen was filled with ground cells when cultured on *E. coli* **(A-D)**. Intestinal lumen had intact cells when nematodes were cultured on *P. luminescens* **(E-H)**. Arrows indicate the terminal bulb. Anterior is left. Scale bars, 50 μm.

*C. elegans* ground *E. coli* in the terminal bulb, digested them and absorbed the nutrition in the intestinal cells (Figure 3A-D). However, *C. elegans* couldn’t grind the *P. luminescens*, intact bacterial cells remained but the bacteria did not proliferate in the intestinal lumen (Figure 3E-H).

### Two pathways influence *C. elegans*resistance to *P. luminescens*

Given that the insulin/IGF-1 signaling pathway and the p38 MAPK pathway in *C. elegans* are required for the defense response against several bacterial infections in intestine (Tan and Shapira 2011), we examined the role of these pathways against *P. luminescens*. The bZIP transcription factor ATF-7 is phosphorylated by the MAP kinase PMK-1 (Shivers et al. 2010), which activates numerous antimicrobial enzymes and peptides such as LYS-2 and CLEC-67 (Troemel et al. 2006). When the *pmk-1* was knocked down by RNAi, *C. elegans* lifespan decreased significantly (*P* < 0.005) (Figure 1A, Table 1). The FOXO transcription factor DAF-16 is negatively regulated under the control of the insulin/IGF-1 receptor DAF-2 (Murphy et al. 2003), regulating the expression of antimicrobial enzymes and peptides such as SPP-1, LYS-7 and THN-2 (Murphy et al. 2003; Evans et al. 2008). Knockdown and null mutation of *daf-16* did not affect *C. elegans* lifespan (*P* > 0.05) (Figure 1B, D, Table 1). On the other hand, *C. elegans* lifespan significantly increased when *daf-2* was knocked down (*P* < 0.005) (Figure 1C, Table 1). Moreover, knockdown of *daf-2* didn’t extend lifespan in *daf-16* null mutant (Figure 1D, Table 1). The size of crystal-like structure was not affected by these pathways (data not shown).

### *P. luminescens*suppresses some *daf-16*-regulated antimicrobial genes in *C. elegans*

**Figure 4:**
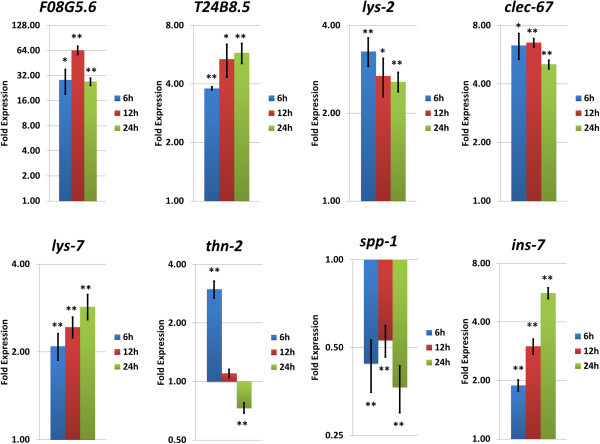
**Relative gene expression changes of the seven antimicrobial enzymes and peptides, and** ***ins-7*** **genes in** ***C. elegans*** **, fed on** ***P. luminescens*** **for 6, 12 and 24 hours.** Values are means ± SE from at least three independent experiments. The significant differences were determined by Student’s t-test, compared to a normalized value of 1.0 for control *C. elegans* fed on *E. coli* OP50. *P < 0.05; **P < 0.005.

Next, we tested the expression of seven enzymes and peptides which are thought to work against microbial infection under the controls of these insulin/IGF-1 and p38 MAPK pathways after 6, 12 or 24 hours of *P. luminescens* exposure. We found that all four p38 MAPK-regulated genes, F08G5.6, T24B8.5, *lys-2*, and *clec-67*, were induced in each time point (Figure 4). The insulin/IGF-1-regulated genes, however, were differentially expressed. *lys-7* was induced and *spp-1* was suppressed in all time points; *thn-2* was induced at 6 hours, unchanged at 12 hours, then repressed at 24 hours (Figure 4). The expression of one of the insulin/IGF-1 peptides, INS-7, a ligand of DAF-2 which suppresses DAF-16 activity (Murphy et al. 2003; Kawli and Tan 2008), was induced at all time points (Figure 4).

**Figure 5:**
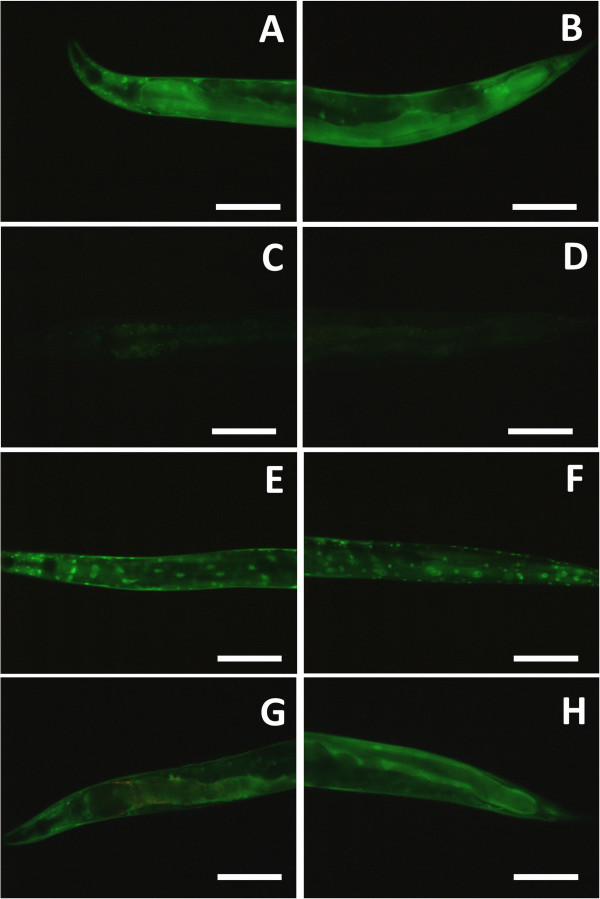
**DAF-16::GFP does not respond to** ***P. luminescens*** **. (A, B)** DAF-16::GFP was located in cytosol when *C. elegans* was grown on *E. coli* OP50. **(C, D)** Cytosolic DAF-16::GFP was diminished when *daf-16* was knocked down. **(E, F)** Cytosolic DAF-16::GFP was accumulated into the cell nuclei when *daf-2* was knocked down. **(G, H)** DAF-16::GFP translocation was not seen when *C. elegan*s was exposed to *P. luminescens* TT01. Scale bars, 100 μm.

Several external stresses, such as starvation, heat, and oxidative stresses induce DAF-16 translocation from cytosol to nucleus, inducing several stress responsive and antimicrobial peptides (Henderson and Johnson 2001). Depletion of *daf-16* clearly diminished cytosolic DAF-16::GFP (Figure 5A-D). When *daf-2* was knocked down, DAF-16::GFP was accumulated into nuclei (Figure 5E, F). Exposure to *P. luminescens* TT01 fails to promote DAF-16::GFP translocation (Figure 5G, H).

### *P. luminescens*partially suppresses *C. elegans*resistance by INS-7 induction

**Figure 6:**
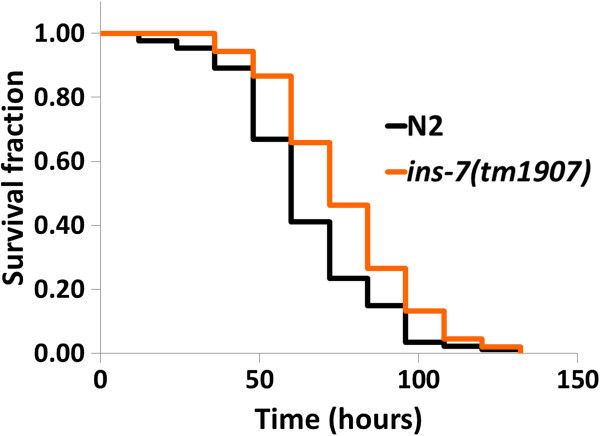
**Deletion** ***ins-7*** **mutant significantly increased lifespan.** Deletion *ins-7*(*tm1907*) mutant increased resistance against *P. luminescens* (*P* < 0.001). Survival curves are presented based on two trials (see Table 1). *P* values were calculated by log-rank test compared with the N2.

Some *daf-16*-regulated antimicrobial genes were suppressed, and one of the *daf-2* ligand *ins-7* was induced by *P. luminescens*, given that *P. luminescens* may suppress *C. elegans* resistance by *ins-7* inductions. We next examined if the loss of *ins-7* function would not suppress *C. elegans* resistance and extend life span. The deletion *ins-7*(*tm1907*) mutant significantly increased lifespan (Figure 6, Table 1), suggesting that *P. luminescens* partially suppresses *C. elegans* resistance by INS-7 induction.

## Discussion

We showed that when *C. elegans* was fed on *P. luminescens* TT01, intestinal cells became delicate and eventually collapsed (Figure 2C-D). Some insecticidal toxins such as Tc and Mcf produced by *P. luminescens* W14 were reported to cause destructions of midgut epithelium in insects (Bowen et al. 1998; Daborn et al. 2002). When *C. elegans* was fed on the recombinant *E. coli* expressing the *P. asymbiotica mcf1* gene, severe feeding delay was observed (Waterfield et al. 2008) suggesting the possibility of damage on nematodes intestine. And a toxin complex TcaA was necessary but not sufficient for *C. elegans*-killing ability in *Yersinia enterocolitica* (Spanier et al. 2010). Such possible toxins produced by *P. luminescens* might be responsible for the intestinal cell deformation.

To our knowledge, there is no report about the formation of crystal-like structure in nematode intestinal lumen followed by bacterial infection including other *Photorhabdus* strains. *P. luminescens* is reported to produce two types of intracellular protein inclusions, CipA and CipB, which support growth and reproduction of the mutualistic EPN but have no effect on insect pathogenesis (Bintrim and Ensign 1998). Because Cip proteins are so small in size and produced inside of the bacterial cell, these inclusion proteins might be different from the crystal-like structure in the present study. The crystal-like structure still remains in *C. elegans* intestinal lumen after transferred onto *E. coli* lawn and their growth and reproduction are resumed (Additional file 2, Table 2); this crystal was unlikely to be toxic for *C. elegans*.

Many pathogenic bacteria colonize and proliferate in the intestinal lumen of *C. elegans* to exert their pathogenicity (Tan et al. 1999; Garsin et al. 2001; Kurz et al. 2003). Our result clearly showed that *P. luminescens* was not ground up by *C. elegans* terminal bulb, and remained intact into the intestinal lumen without proliferation. This may suggest that *P. luminescens* adopts a different strategy to kill nematodes, for example, via production of toxins without colonization.

Four antimicrobial genes (F08G5.6, T24B8.5, *lys-2*, *clec-67*) under the control of the p38 MAPK pathway, were up-regulated when fed on *P. luminescens* for 6, 12 and 24 hours (Figure 4). Up-regulation of these genes was also observed when infected by other pathogenic bacteria e.g. Gram-negative bacterium *Pseudomonas aeruginosa* (Troemel et al. 2006; Evans et al. 2008; Shivers et al. 2010). In addition, when *pmk-1* was knocked down by RNAi, *C. elegans* became more susceptible to *P. luminescens* (Figure 1A, Table 1), which suggests that this pathway contributed resistance against *P. luminescens* but didn’t affect enough for survival. This pathway is reported to act in a cell-autonomous manner in intestine to regulate innate immunities against bacterial infections (Kim et al. 2002; Troemel et al. 2006; Shivers et al. 2009, 2010; Engelmann et al. 2011).

On the other hand, three antimicrobial genes (*spp-1*, *thn-2*, *lys-7*) regulated by the insulin/IGF-1 signaling pathway showed different expression patterns when fed on *P. luminescens* (Figure 4). This pathway is also related to host defense against several pathogens (Garsin et al. 2003; Murphy et al. 2003), and its activation is regulated in a non-cell-autonomous manner by secretion of INS-7 from the nervous system, which negatively regulates infection-related genes expression via the transcription factor DAF-16 (Kawli and Tan 2008; Tan and Shapira 2011). Infection of *P. aeruginosa* PA14 suppressed the expression of these three genes through the induction of INS-7 expression (Evans et al. 2008). However, transcriptional analysis using tiling arrays and RNA-sequencing showed the *spp-1* and *thn-2* were down-regulated, but *lys-7* and *ins-7* were not affected when *C. elegans* was fed on *P. luminescens* strain Hb (Engelmann et al. 2011).

We couldn’t observe translocation of DAF-16::GFP into cell nuclei after *P. luminescens* infection (Figure 5). As shown in our experiments, resistance of *C. elegans* against *P. luminescens* was increased when DAF-16 was activated by *daf-2* RNAi (Figure 1C-D, Table 1). However, DAF-16 was not activated by the bacteria, which is consistent with the fact that *daf-16* RNAi did not significantly decrease *C. elegans* lifespan on *P. luminescens* (Figure 1B, Table 1).

Up-regulation of *ins-7* and the importance of *ins-7* for DAF-16 nuclear delocalization during the infection of *P. aeruginosa* to *C. elegans* have been reported (Evans et al. 2008). *P. luminescens* induced overexpression of INS-7 in *C. elegans* (Figure 3), which might keep DAF-16 in the cytoplasm via the insulin/IGF-1 signaling pathway. This mechanism seems unique to *P. luminescen*s and *P. aeruginosa*. Gram-positive bacterial pathogen *Enterococcus faecalis* V583 induces the DAF-16-controlling genes in *C. elegans* (Evans et al. 2008), and Gram-negative bacterium *Salmonella typhimurium* induces *spp-1* in *C. elegans*, which contribute to suppress bacterial proliferation in the intestine (Alegado and Tan 2008). Although *P. luminescens* and *P. aeruginosa* suppress the nematode immune response in the same way, they don’t share their natural hosts. They might have developed the immune-suppressive mechanism independently.

## Conclusions

We showed here the high pathogenicity of the EPN mutualistic bacterium *P. luminescens* TT01 to the model organism *C. elegans*. The insulin/IGF-1 signaling pathway is highly effective for the resistance against *P. luminescens*, albeit DAF-16 is inactivated by the bacteria via INS-7 induction, which was also reported in human opportunistic pathogen *Pseudomonas aeruginosa* PA14. This infection strategy of *P. luminescens* might also be applied to infect insects.

## Methods

### Nematodes and bacteria strains and culturing

*C. elegans* and Rhabditidae sp. culturing and handling were performed as described previously (Brenner 1974), except when otherwise noted. Strains used in this study were: *Caenorhabditis elegans* N2 (Bristol strain), TJ356 *{zIs356[Pdaf-16::daf-16::gfp, pRF4]IV}* (Henderson and Johnson 2001), CF1038 *daf-16(mu86)I* (Lin et al. 1997), *ins-7(tm1970)* (provided by the National BioResource Project, Japan), Rhabditidae sp. KHA410. *Heterorhabditis bacteriophora* TT01 (kindly provided by Ann Burnell, National University of Ireland-Maynooth) was maintained by infecting the greater wax moth *Galleria mellonella* larvae as following the methods described previously (Kaya and Stock 1997). The symbiotic bacteria *P. luminescens* TT01 was isolated from *G. mellonella* infected with *H. bacteriophora* TT01, spread on MacConkey agar plates to select the primary phase colonies. The primary phase *P. luminescens* TT01 colony was picked up, transferred into LB media and grown overnight at 28°C with 200 rpm shaking. About 200 μl or 400 μl of the liquid cultured bacteria was spread on a 6 cm or 9 cm NGM plate, respectively, without clearance space, incubated 32 hours at 28°C, and then cooled down to 20°C. All fresh *P. luminescens* TT01 plates were prepared just before use.

### Construction of *gfp*-labeled bacteria

GFP-labeled *E. coli* OP50 and *P. luminescens* TT01 were constructed by the miniTn7-transposon system pBK-mini*Tn*7-*gfp*2 and pUX-BF13 (Lambertsen et al. 2004; Easom et al. 2010). Briefly, *E coli* S17-1 (λ pir) carrying pBK-mini*Tn*7-*gfp*2 (donor) and *E. coli* SM10 (λpir) carrying pUX-BF13 (helper), and *E. coli* OP50 rifampicin resistance (Rif^R^) or *P. luminescence* TT01 Rif^R^ (recipients) growing in LB medium containing 10 mM MgCl_2_ with appropriate antibiotics (MgLB) (OD_600_ = 0.5) were mixed in a 1:1:4 ratio (donor: helper: recipient = 50 μl: 50 μl: 200 μl), dropped on the center of MgLB agar plate and then incubated overnight at 30°C. Thereafter, bacterial cells were collected with MgLB and plated on MgLB agar containing 50 μg/ml rifampicin and 5 μg/ml chloramphenicol, and incubated for 48 hours at 30°C. Correct position of the *Tn*7 insertion in the colonies acquired were confirmed by PCR with the primers, Tn7-Pl_glmS, 5’ – CGT AAT TTG GCT AAA TCA GTG AC – 3’ and Tn7R109, 5’ – CAG CAT AAC TGG ACT GAT TTC AG – 3’.

### Survival test

All survival tests were carried out at 20°C. Synchronized L1 stage *C. elegans* were obtained by treating egg-containing adults with sodium hypochlorite (Porta-de-la-Riva et al. 2012) and allowed to grow on NGM plates seeded with *E. coli* OP50 at 20°C for 48 hours until the late L4 stage. L4 stage *C. elegans* were washed with M9 buffer and transferred onto the 6 cm NGM plates completely covered with *P. luminescens* TT01, and checked every 12 hours until nematode death. Preparation of the NGM plate seeded with fresh *P. luminescens* TT01 was described as above. The time point of nematode transfer onto the *P. luminescens* plate represented the zero hour of life-span analysis. Nematodes began to lay a few eggs after about 12 hours, but the hatched larvae couldn't grow and were easily distinguish from their parents. Nematode was considered dead when it no longer responded to light prodding with a platinum wire. Nematodes that crawled-up to the dish wall and desiccated were excluded from the data. Experiments were performed two or three times. Survival curves were analyzed by the Kaplan-Meier procedure, and significant differences between survival curves were calculated by the log-rank test with statistical software Excel Tokei 2006 (SSRI, Tokyo, Japan).

### Formation of crystal-like structure

Synchronized L4 stage *C. elegans* were transferred onto the 9 cm NGM plates completely covered with *P. luminescens* TT01, prepared as described above, and incubated at 20°C for 12 hours. Then nematodes were collected and washed with M9 buffer twice and transferred onto the 9 cm NGM plates seeded with *E. coli* OP50, and incubated at 20°C for 24 and 48 hours. After 24 or 48 hour incubation, 10–20 nematodes were randomly picked up and observed the formations of crystal-like structure with the differential interference contrast (DIC) microscopy. Each observation was performed three times independently.

### RNA interference (RNAi)

Gene fragments of *C. elegans daf-2*, *daf-16* and *pmk-1* were prepared by PCR amplification of *C. elegans* N2 cDNA and cloned into the RNAi vector pPD129.36 (kindly provided by Fire, A., Stanford University). The PCR fragment-ligated plasmid or the blank vector pPD129.36 was used to transform *E. coli* HT115 (Kamath et al. 2001). Primers for RNAi constructs were listed in Table 3. For RNAi experiments, synchronized L1 stage nematodes were cultured until L4 stage for 48 hours at 20°C on NGM (containing 50 μg/mL ampicillin and 20 μg/mL tetracycline) plates seeded with *E. coli* HT115 transformed with each different RNAi plasmid. Synchronized L4 stage nematodes were then collected and transferred onto survival test plates.

**Table 3:** **Primers used for RNAi constructs」**

	Primer name	Sequence
1.	daf-16a_BamHI_For	5’ – GGGGATCCGCCGGAGCCACGTGGCAGGTG – 3’
2.	daf-16a_SalI_Rev	5’ – ATACGCGTCGACTCAGCTCATGTCTGATCAATG – 3’
3.	daf-2_SalI_For	5’ – GGAGCACGATATTGTCGACGGCA – 3’
4.	daf-2_XbaI_Rev	5’ – GCTCTAGATTTCTGAACAGTGACTTTGCCT – 3’
5.	pmk-1_EcoRI_For	5’ – GGGAATTCCACAGACAACAATGGATCATAT – 3’
6.	pmk-1_EcoRI_Rev	5’ – AAGGAATTCTTCATCTGGTGTTCC – 3’

### Feeding test

Synchronized L4 stage *C. elegans* were washed with M9 buffer and transferred onto the 6 cm NGM plates completely covered with *gfp*-tagged *P. luminescens* TT01, or *E. coli* OP50. Nematodes were picked up at every time point and observed with the Nikon E600 microscope equipped with Nomarski DIC system and a fluorescence filter.

### Quantitative RT-PCR

Gene expression of *spp-1*, *thn-2*, *lys-7* (reported to be controlled by the insulin/IGF-1 signaling pathway, Murphy et al. 2003; Evans et al. 2008), F08G5.6, T24B8.5, *lys-2*, *clec-67* (reported to be controlled by the p38 MAPK pathway, Troemel et al. 2006) and *ins-7* (an insulin/IGF-1 peptide and DAF-2 ligand) were analyzed by qRT-PCR using SYBR® green assay (Takara bio, Shiga, Japan). Synchronized L4 stage *C. elegans* grown on the NGM seeded with *E. coli* OP50 were collected, washed with M9 buffer and transferred onto the 9 cm NGM plates completely covered with *P. luminescens* TT01 (prepared as described above) or *E. coli* OP50, and grown at 20°C. After 6, 12 or 24 hours, nematodes were collected, washed twice with M9 buffer, then immediately frozen in liquid nitrogen, and stored in −80°C freezer. Total RNA was extracted by the RNeasy® Plus Micro Kit (Qiagen, Venlo, Netherland) following the manufacturer’s protocol. Total RNA (adjusted for concentration of 50 ng/μl) was reverse transcribed using Oligo dT primer and PrimeScript RT reagent Kit (Takara bio). Quantitative RT-PCR was performed using SYBR® Premix Ex Taq™ II (Tli RNaseH Plus) (Takara bio) on CFX96™ Real-Time PCR Detection System (Bio-Rad, Berkeley, CA, USA). Primers were designed using Primer 3 software (http://simgene.com/Primer3) and tested for specificity prior to qRT-PCR. The housekeeping *snb-1* gene was used as an internal control gene for calculation of relative expression levels of each gene. Primers for qRT-PCR were listed in Table 4. A single peak at the melting temperature of the PCR-product confirmed primer specificity. Experiments were performed at least three times using independent nematode samples. Relative gene expression of each gene was analyzed using ^ΔΔ^CT method (Livak and Schmittgen 2001).

**Table 4:** **Primers used for qRT-PCR analysis**

	Primer name	Sequence
1.	F08G5.6_For	5’ – ATCGTTCCGAATGGTGGTTGAC – 3’
2.	F08G5.6_Rev	5’ – GCCGATTTCAGCTTGCAAAGTG – 3’
3.	T24B8.5_For	5’ – AAACCTGTGGTGTCTGCGTTAC – 3’
4.	T24B8.5_Rev	5’ – TGGCAGGTTTTTGGGCATTG – 3’
5.	lys-2_For	5’ – TGCTGATTTCCGTGCTTTCG – 3’
6.	lys-2_Rev	5’ – TTCCAACAGCATACACGTCACG – 3’
7.	clec-67_For	5’ – AATGTTCAATCGGCCACCCTTG – 3’
8.	clec-67_Rev	5’ – TGGTCATGTTGAAGACGTTCGC – 3’
9.	spp-1_For	5’ – TTTGCTGGACTATGCTGTTGCC – 3’
10.	spp-1_Rev	5’ – AACATCCTTGCACGCCTTGTC – 3’
11.	thn-2_For	5’ – TCCAACTTACGGCTGGACAATC – 3’
12.	thn-2_Rev	5’ – TGCATTGCTCCGAGTTTCTGC – 3’
13.	lys-7_For	5’ – AATGTGCCGTCAAACTTGGC – 3’
14.	lys-7_Rev	5’ – TGCACGAACGAAAACTGCAC – 3’
15.	ins-7_For	5’ – TTAGGTCCAGCAGAACCAGAAG – 3’
16.	ins-7_Rev	5’ – CGCATGCTTTTCCACAAACCG – 3’
17.	snb-1_For	5’ – TGGAGCGTGATCAGAAGTTGTC – 3’
18.	snb-1_Rev	5’ – TCCACCAATACTTGCGCTTCAG – 3’

## Authors’ information

JSPS Research Fellow.

## Electronic supplementary material

Additional file 1: **Crystal-like structure is also constructed in another bacteriovorous nematode.** L4 stage Rhabditidae sp. was cultured on *P. luminescens* for 24 hours at 25°C. Arrow indicates crystal-like structure inside the intestinal lumen. (JPEG 589 KB)

Additional file 2: **Crystal-like structures do not disappear once constructed.** (A) Frequency of the crystal-like structure construction. There is no significant difference between before and after culturing on *E. coli* OP50 (*P* > 0.05, Fisher’s exact test). Integrated data of three independent experiments are shown. (B) *C. elegans* body shape, grown on *E. coli* for 24 hours after 12-hour incubation on *P. luminescens* from the L4 stage. Scale bar, 200 μm. (C) Crystal-like structure in the *C. elegans* intestine, grown on *P. luminescens* for 12 hours from the L4 stage. Scale bar, 50 μm. (D) Crystal-like structure in the *C. elegans* intestine, grown on *E. coli* for 48 hours after 12-hour incubation on *P. luminescens* from the L4 stage. Arrows indicate crystal structure. Scale bar, 50 μm. (JPEG 777 KB)
