# Supplementary figures and images for: Activated and inactivated immune responses in Caenorhabditis elegans against Photorhabdus luminescens TT01
Source: Springerplus. 2014 Jun 1;3:274. doi: 10.1186/2193-1801-3-274 (PMC4171960; doi:10.1186/2193-1801-3-274)

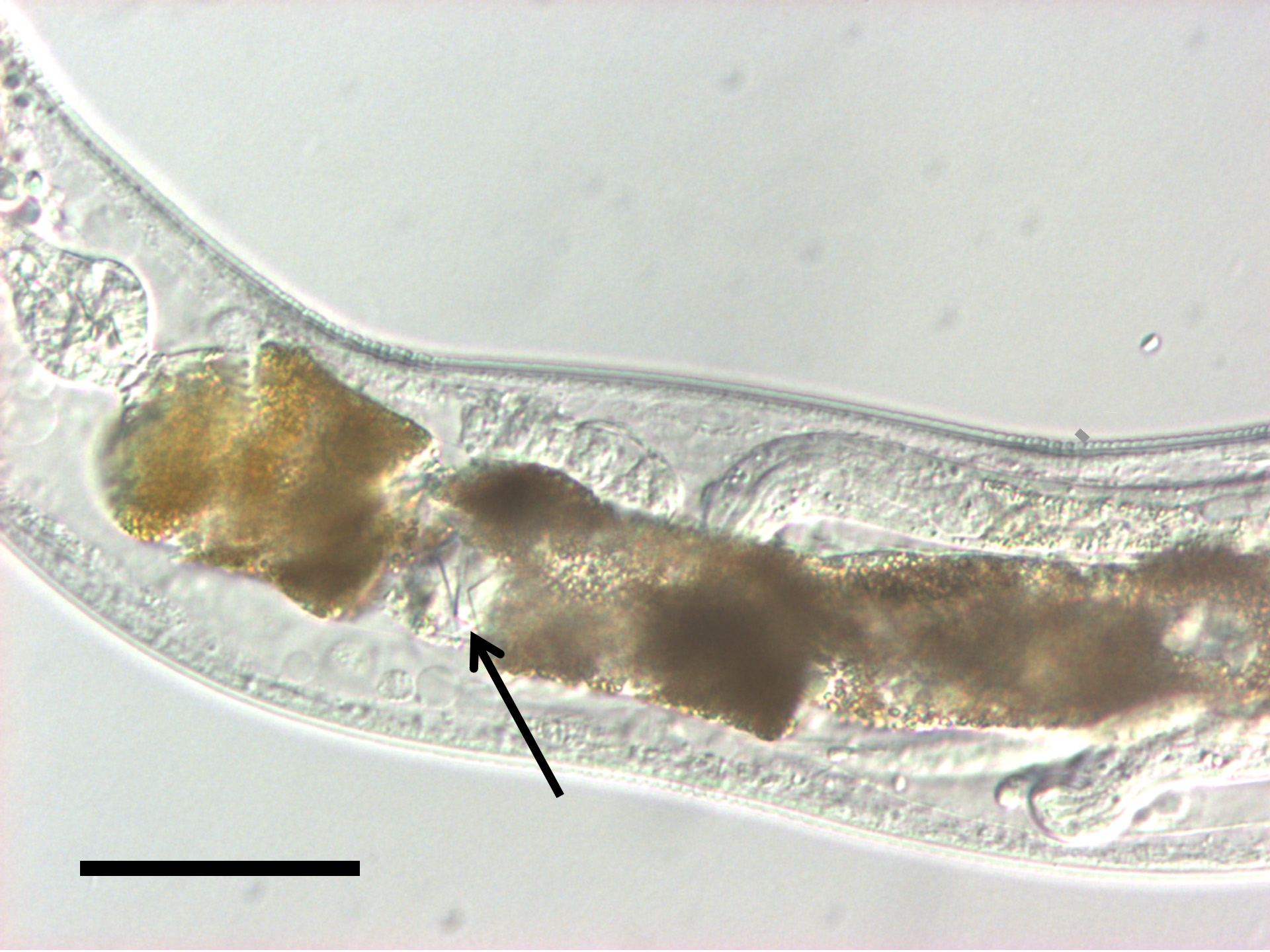

Supplement: Supplementary file 1 — Additional file 1: Crystal-like structure is also constructed in another bacteriovorous nematode. L4 stage Rhabditidae sp. was cultured on P. luminescens for 24 hours at 25°C. Arrow indicates crystal-like structure inside the intestinal lumen. (JPEG 589 KB) [file 40064_2013_1013_MOESM1_ESM.jpeg]

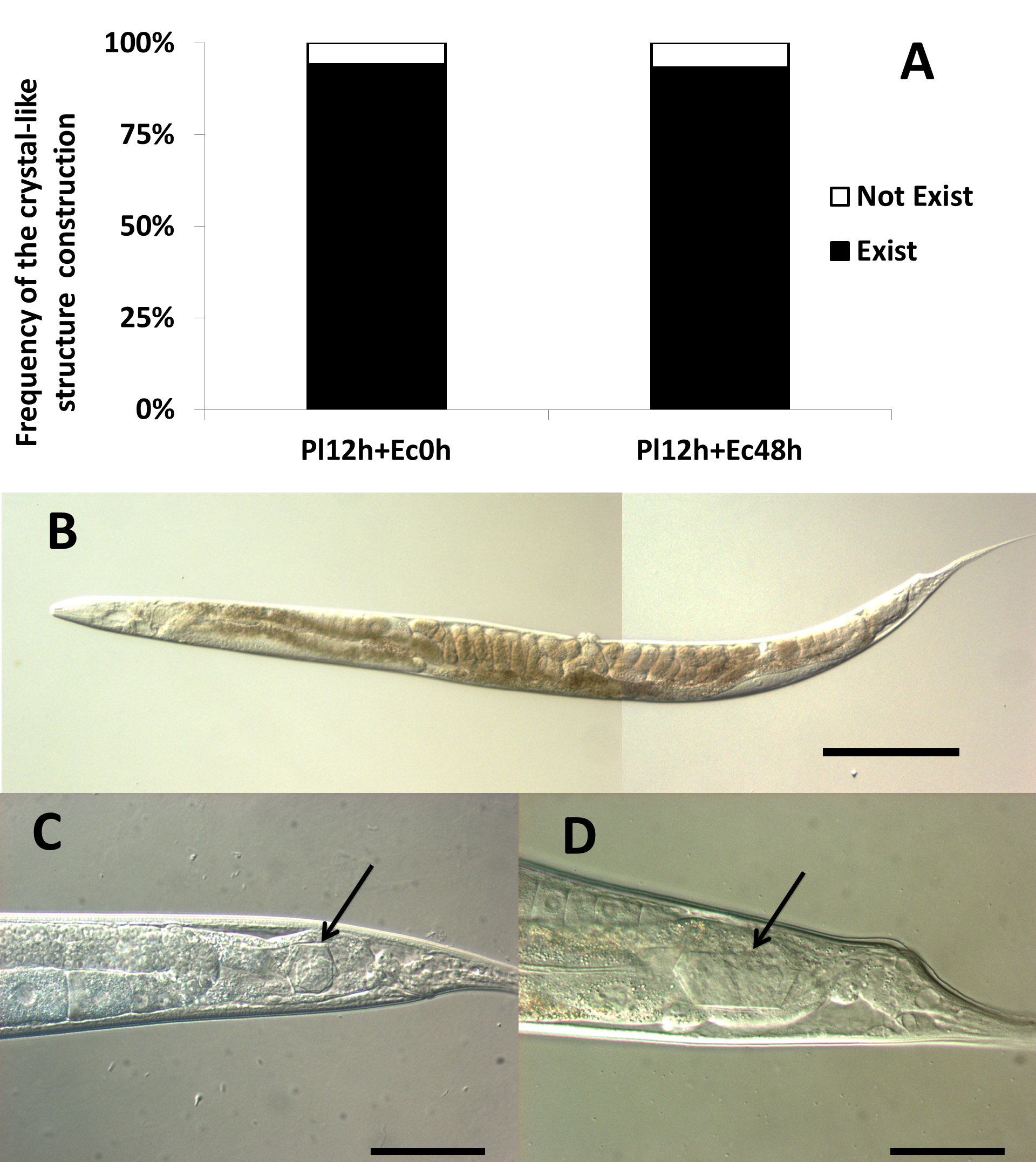

Supplement: Supplementary file 2 — Additional file 2: Crystal-like structures do not disappear once constructed. (A) Frequency of the crystal-like structure construction. There is no significant difference between before and after culturing on E. coli OP50 (P > 0.05, Fisher’s exact test). Integrated data of three independent experiments are shown. (B) C. elegans body shape, grown on E. coli for 24 hours after 12-hour incubation on P. luminescens from the L4 stage. Scale bar, 200 μm. (C) Crystal-like structure in the C. elegans intestine, grown on P. luminescens for 12 hours from the L4 stage. Scale bar, 50 μm. (D) Crystal-like structure in the C. elegans intestine, grown on E. coli for 48 hours after 12-hour incubation on P. luminescens from the L4 stage. Arrows indicate crystal structure. Scale bar, 50 μm. (JPEG 777 KB) [file 40064_2013_1013_MOESM2_ESM.jpeg]
